# Seasonal variations in measurements of linear accelerator output

**DOI:** 10.1002/acm2.12548

**Published:** 2019-02-28

**Authors:** Steven Bartolac, Robert Heaton, Bernhard Norrlinger, Daniel Letourneau

**Affiliations:** ^1^ Radiation Medicine Program Princess Margaret Cancer Center Toronto ON Canada; ^2^ Radiation Oncology Johns Hopkins School of Medicine Baltimore MD USA

**Keywords:** dosimeter, humidity, linear accelerator, quality assurance, quality control, seasonal

## Abstract

**Purpose:**

Seasonal trends in linear accelerator output have been reported by at least one institution and data have suggested that they may be present at our center as well. The purpose of this work was to characterize these trends and determine whether local environmental conditions within the treatment rooms may be impacting the linear accelerators and/or the quality control (QC) dosimeter.

**Methods:**

Runtime plots of daily output data, acquired using an in‐house ion chamber‐based device, over 3 yr and for 15 linear accelerators of different makes and models were reviewed and evaluated. Environmental conditions were monitored prospectively in a representative treatment room for approximately 9 months and evaluated for correlations with output trends. Independent measures of output using daily MV portal images were compared with output measurements using the ion chamber‐based device. A separate controlled experiment probing the response of the in‐house dosimeter to humidity changes over time was also carried out using a constant current source and a small enclosure.

**Results:**

Runtime plots of output revealed sinusoidal, seasonal variations that were consistent across all treatment units, irrespective of manufacturer, model, or age of machine. The amplitude of the variation was on the order of 1% and maintained a yearly period. The independent measure of output using MV portal images did not corroborate the seasonal trends observed with the daily QC dosimeter. Based on the controlled experiment, the QC dosimeter was found to have a dependence on relative humidity changes, decreasing 1% in output per 30% increase in relative humidity.

**Conclusions:**

Results confirm the presence of underlying seasonal variations in measured output from the linear accelerators. The findings identify humidity impact on the measurement device as the underlying cause of the cyclical changes and not the accelerators themselves. These results could help minimize unwarranted machine servicing.

## 1 INTRODUCTION

Daily quality control (QC) tests for linear accelerator output have been recommended by most national guidelines in North America and Europe.^1^, ^2^ Output measurements that exceed tolerance levels require investigation, create delay in patient treatment, and may require adjustments to restore functionality of the equipment to within tolerance. Seasonal variations in output measurements have been observed and reported in the literature by at least one center.^3^ That center's report suggests that the variations observed may be related to both the measurement device and the accelerator itself. The report also suggests that humidity may be the underlying cause of the observed behavior, though further investigation into causative factors was not explored.

Output levels at Princess Margaret Cancer Centre (PMCC) have been observed on various accelerators of different make and model to drift over time. The nature of these drifts has been noted in some cases to be positive for several months followed by a negative drift thereafter. These observations led to the suspicion that seasonal variations may exist on one or more units at PMCC as well and prompted investigation.

The aim of this paper was to evaluate seasonal and/or other trends that may exist in output measurements of one or more linear accelerators at our center. Additionally, we also investigated potential environmental factors that may correlate to such changes and whether observed changes are related to actual changes in output of the accelerator or systematic errors in the measurement device or both.

## 2 MATERIALS AND METHODS

Seasonal variations were analyzed through a review of 6 MV photon output measurements available for 15 active linear accelerator units. We note that the accelerators at our center are not exclusive to one manufacturer and models include both sealed and unsealed monitor unit chamber types. The retrospective data review spanned approximately 3 yr; a shorter period of review was utilized in the case of three units that were less than 3 yr old, and for two of the 15 units that were decommissioned before the end of the review period. On commencement of the study, data were collected prospectively alongside environmental monitoring of selected units for approximately 9 months.

Our center utilizes an internally developed ion chamber array (49 top‐hat type ion chambers) for monitoring accelerator daily output.^4^ Only the central chamber (aligned with the central axis of the treatment unit) is used for output measurement, while the remaining chambers are utilized for symmetry and flatness measurements. Each treatment room is equipped with its own ion chamber array which we will refer to herein as the PMCC Matrix.

### 2.A Service events and data processing

Outputs from linear accelerators have been observed at our center to change on the order of up to a few percent during initial months post commissioning and before stabilizing thereafter. Where such variations were identified, the initial data points were excluded from the analysis.

Characterization of trends in output data is challenged by occasional output adjustments made to linear accelerators and/or recalibration of the PMCC Matrix, which introduces step discontinuities in the measured data (typically, a change of about 2%). These events are referred to as *service events*. In a manner similar to that described by Hossain et al.^3^, we attempt to represent the machine output as a function of time with the impact of these servicing events removed from the data. In order to simulate this scenario, the bias introduced at each service event is first estimated as the difference in average reading (over five data points) prior to and following the event; this bias is then removed via subtraction to all data points following the event. Note that the PMCC Matrix is cross calibrated with absolute farmer chamber measurements such that a reading of unity corresponds to the machine output at reference conditions. In the case where the bias was a result of a change to the calibration factor of the PMCC Matrix, the data were corrected to the prior known calibration factor rather than using the above subtraction estimate.

An additional processing step was introduced for instances where nonperiodic trends were observed in the data. Such trends are characterized by clear monotonic increases in output and have been associated with leaks in sealed monitor unit chambers on some units. These trends were modeled using a least squares linear regression and then removed by subtraction in order to better observe any overlaying periodic trends present in the data.

Service events were identified by review of service logs (visual inspection). In cases where the drift from baseline was greater than or equal to 1% prior to the service event and restored to baseline following it, it was suspected that the output drift itself was the main trigger or indicator of service being required. The number of such cases was recorded and compared to the total number of service events identified.

### 2.B Environmental monitoring

Environmental conditions in a representative treatment unit were monitored using two SD700 Dataloggers (Extech, Townsend West, NH), capable of monitoring pressure, temperature, and relative humidity levels. Pressure and temperature measurements using these *dataloggers* were cross calibrated with a NIST traceable reference barometer (Setra Model 470 digital pressure transducers, Boxborough, MA) and thermometer (Omega HH40 thermistor thermometer, Omega Engineering, Stamford, CT) prior to use. Reference hygrometers are not readily available at our center. The assumed accuracy of the relative humidity measurements was ±5% as specified by the manufacturer. Cross reference between dataloggers suggested agreement on the order of 2% between the devices. The treatment unit investigated was arbitrarily selected, and in this case was an Elekta Agility unit commissioned in spring 2013. This unit will be referenced as unit G in this manuscript.

#### 2.B.1. Pressure and temperature

Pressure readings used for ion chamber temperature–pressure correction factors are acquired from a barometer placed in a laboratory on the same floor as that of the treatment units (readings available online, updated every 5 min). Given that a single barometer reading is utilized for correction factors for all units, it was hypothesized that machine output seasonal variations may be a result of systematic and periodically varying differences between the pressure recorded in the laboratory and that measured within the treatment units. Pressure information was, therefore, acquired from a representative treatment unit and compared to the barometer for consistency.

Historical data observed suggested a reasonable time interval over which we would expect to see maximum variation is between the months of February and September. Data were therefore collected over an approximately 9‐month period beginning in February, with readings collected at 5 min intervals. Data comparison was done by interpolating the treatment unit data to the same time points as the laboratory barometer and observing the difference between the datasets as a function of time. A seasonal drift in the reported pressure of approximately 1 kPa would be required to generate 1% artifact in the measured unit output.

Each treatment unit is equipped with its own individual thermometer placed on the treatment unit wall, which is used to record the room temperature at the time of output readings. When the ion chamber array used for daily measurements is not in use, it is generally stored in a designated cupboard within the room; it was thought that a temperature gradient between the wall and the storage area might have an associated seasonal variation leading to erroneous correction factors. This hypothesis was tested by measuring the temperature within the storage area and comparing it to the measurements collected on the wall; temperature data were collected in conjunction with the pressure readings described above and at the same time intervals. Consistent temperature differentials on the order of 3 degrees would need to be observed for a systematic error in output measurement of approximately 1% to exist due solely to temperature inaccuracies. The combined pressure temperature correction factor was also monitored for any systematic or seasonal variation compared with those recorded for the treatment units at time of daily output measurement.

#### 2.B.2. Humidity

Humidity is a factor that is typically neglected in terms of corrections to ion chamber readings since it is well documented^5^, ^6^, ^7^ that changes in humidity in air within the range of 20%–80% are expected to introduce less than 0.15% error. Notwithstanding, the stability of electronics and in particular capacitors are known to be affected by varying humidity levels.^8^ Humidity was therefore monitored over the same time frame and intervals in order to determine any correlations in change in humidity vs output.

### 2.C Independent output measurements

An alternative, independent daily measurement was also desired for comparison with the recorded daily output measurements. Within the same treatment room where environmental monitoring was being performed, daily MV portal images were also being acquired for use in jaw and MLC position quality control tests (unrelated to this study). Although the images were not originally being acquired for use in relative output measurements, they had the potential to be used for this purpose since an average signal value over a central region of the MV portal imager is expected to change with accelerator output, provided the changes in signal are above the noise floor of the measurement.

The sensitivity of detector signal to linear accelerator output changes was verified by comparing the signal value before and after a service event that was known to result in an output change on the order of 2%. Historical daily QC data acquired with the portal imager were then analyzed for seasonal trends. The presence of a seasonal variation on one device and not the other would suggest the variations are device dependent and not the result of actual output changes on the unit.

### 2.D Dosimeter response to humidity

A controlled experiment was also performed to analyze the response of the measurement electronics of the PMCC Matrix to humidity. Tests were carried out in a wooden enclosure where environmental conditions could be controlled and monitored. A hole drilled into the enclosure allowed passage of cabling. Once the cables were run through the hole, the remaining space was filled with cloth.

The source of humidity was a tray filled with water placed in the housing. Loose humidifier pads were placed in the tray to help disperse the moisture. Using this method, it was possible to produce humidity levels of up to 85% relative humidity. During the testing period, the ambient relative humidity was approximately 20%. The removal of the pads allowed for testing under the ambient dry conditions. This differential in humidity represented the approximate change between humidity extremes observed within the linear accelerator bunkers over the course of a year.

It was hypothesized that moisture absorption in the capacitor dielectric may affect its relative capacitance. The polyester capacitor of the original Matrix design was, therefore, tested concurrently with two other dielectric materials with different absorption properties for comparison: polypropylene and polyphenylene.

One capacitor of each type was installed in a Matrix dosimeter along separate electrometer channels. The ion chamber connection to the input of each of these channels was removed and replaced by a connection to a current source (Keithley Model 6221, Cleveland, OH). A known 5 nA current was supplied to each electrometer channel. Output of the electrometer was sampled for 30 s using the software's timed integration function. Four samples were acquired and averaged for each measurement on a daily basis over the course of approximately 2.5 months; humidity was increased using the pads after approximately 3 weeks. To verify the output of the current source and to provide a reference measurement, the 5 nA current source output was also measured for 30 s using a commercial electrometer (Fluke 35040). The environmental conditions inside the test chamber were monitored utilizing the datalogger.

## 3 RESULTS

### 3.A Service events and data processing

**Figure 1:**
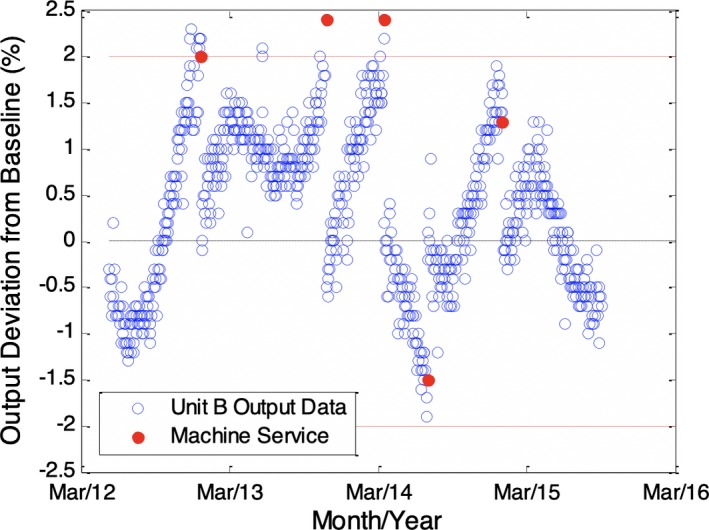
Runtime plot of output measurements from a sample unit. Drifts in output values can be observed in the data to occur in alternating directions, suggesting a variation of periodic nature may be present. Upper and lower dashed lines represent the action tolerance levels where investigation and potential output correction are recommended at our institution.

Figure 1 shows a sample unprocessed dataset of measured output relative to baseline acquisition utilizing the PMCC Matrix, for a 6 MV beam. Solid dots indicate service events that occurred corresponding to either a change in machine output or an adjustment of the PMCC Matrix calibration factor.

**Figure 2:**
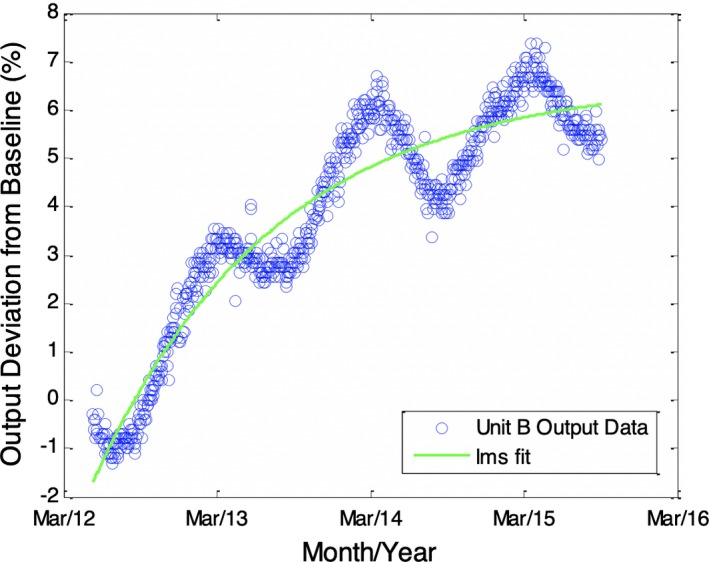
Data from Fig. 1 with step discontinuities in output removed in software to simulate the case if service events had not occurred on the unit. A periodic trend can be seen to overlay an otherwise gradually increasing trend. The increase in output appears to be on the order of 2%–3% per year approaching an asymptote in the most recent months. The data are modeled as a positively increasing trend with exponential decay in growth and using a least mean squares (lms) fit to the data.

**Figure 3:**
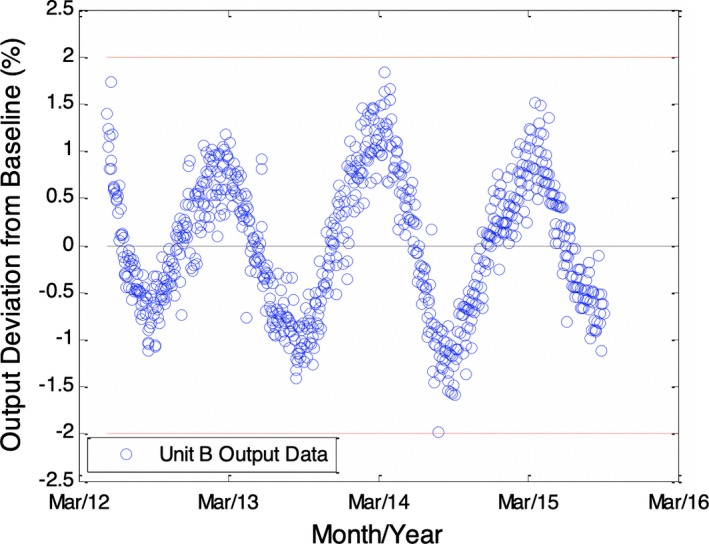
Processed output data shown in Fig. 2 with increasing trend removed from the data. The resulting data show the predominant periodic trend with periodicity of approximately 1 yr.

Figure 2 shows the result of processing the data in Fig. 1 such that the difference before and after service events is estimated and subtracted from the data following the event. The result represents an estimate of the output data that would have been observed in the absence of service events. When processed as shown in Fig. 2, the data are observed to maintain a sinusoidal pattern overlaying an increasing trend. The increase in output appears to be on the order of 2%–3% per year, gradually decreasing and approaching an asymptote for the most recent months; a similar trend was observed for two other units of the same manufacturer. This increase agrees well with other reports^9^, ^10^ on output stability of accelerators. The trend was modeled as an increasing function with exponential decay, where the result is expected to have an asymptote at some maximum value. This situation would be consistent with a leaking monitor chamber that eventually comes to equilibrium with the average ambient pressure; the exponential decay in the increase is also consistent with observations in the literature.^11^ With the trend removed from the data, the seasonal variation appears quite dominant as shown in Fig. 3.

**Figure 4:**
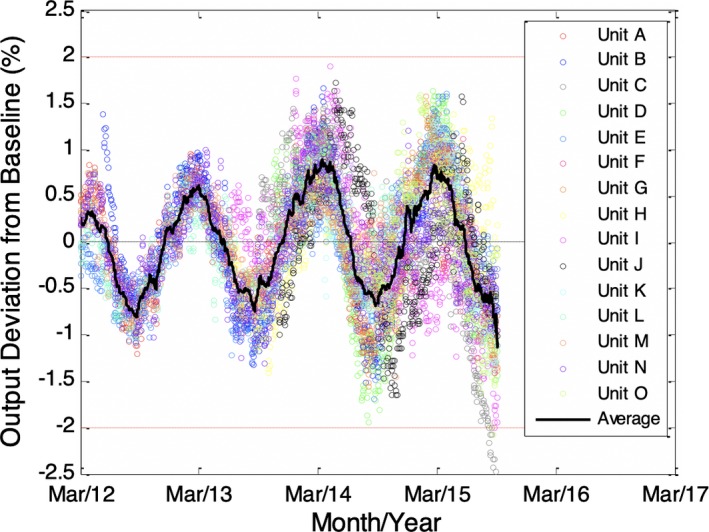
Output with biases due to service events removed and mean values subtracted. Three of 15 units displayed also had increasing trends such as observed in Fig. 2, where the trends were modeled and removed as in Fig. 3: The average periodic trend for all units is also displayed in the graph with amplitude approximately equal to 1% and period equal to approximately 1 yr.

When this processing technique was repeated for all units, a consistent sinusoidal, seasonal variation was observed irrespective of age, model, or make of the units, as shown in Fig. 4. In this figure, the mean of each individual curve has been subtracted. The average curve over all units is also shown. Modeling the average curve as a sinusoid gives an average amplitude of approximately 1% with peaks and valleys occurring in March and September, respectively.

Over the time period reviewed, a combined 43 service events were identified. Of these service events, 27 correlated with the tolerance of 2% being nearly met or exceeded.

### 3.B Environmental monitoring

#### 3.B.1 Pressure and temperature

The standard deviation of the temperature variation between the temperature in the storage location of the QC dosimeter and the ambient temperature within the treatment room was within 0.3°C for the majority of measurements over the period observed. The absolute temperature within the room varied on the order of 2 degrees between spring and fall months. Agreement between pressure measured in the treatment room and the barometer used for temperature pressure correction was within 0.1 kPa. Air pressure readings did not exhibit periodic variation on a seasonal timescale.

#### 3.B.2 Humidity

**Figure 5:**
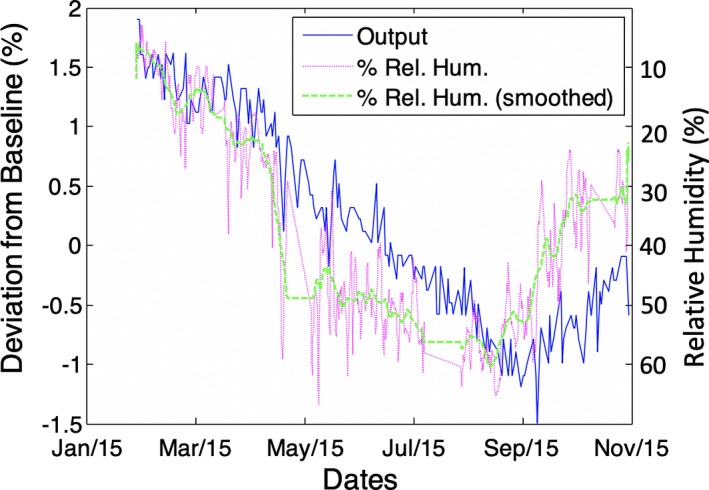
Relative humidity with positive axis down plotted on same graph as relative output measurements for the unit G linear accelerator corresponding to the given treatment room (unit G). The general trend of the humidity is represented by the smoothed curve and correlates well with that of the output data. The trend in output data lags that of the relative humidity by approximately 1 week. Scaling on the graphs was arbitrarily chosen.

Humidity showed an appreciable change over the time period observed, with the lowest humidity recorded near the beginning of the measurement period and the highest humidity occurring in late August. Relative humidity and output showed evidence of anticorrelation, as shown in Fig. 5 where humidity is plotted with positive axis down. The normalized cross correlation coefficient (NCCC) between the smoothed humidity curve and the output data was 0.8 with a lag of the PMCC Matrix data of 1 week. Note that the error bar in the lag is suspected to be large, as much as a week or more, given that local variations in the humidity are still significant in the data even after smoothing (rolling mean smoothing filter with a 10‐day window). Since the humidity was displayed with positive axis down, the NCCC indicates a strong anticorrelation which implies that output measurements drop as humidity increases.

### 3.C Independent measurements

**Figure 6:**
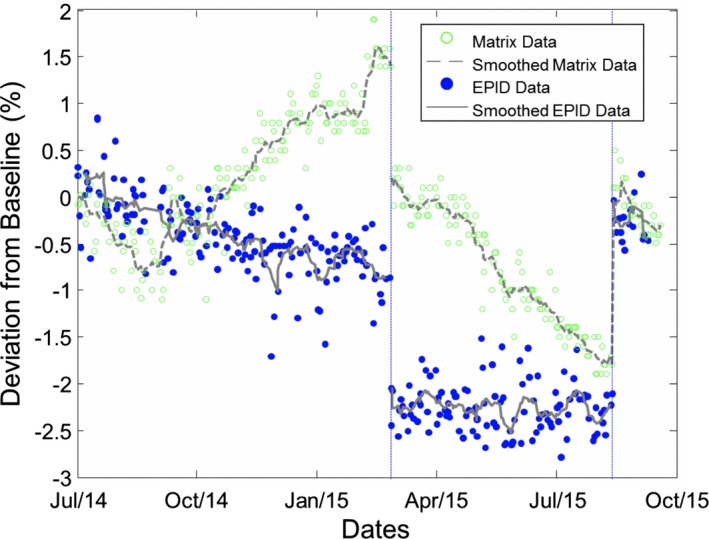
Relative output data measured using the Princess Margaret Cancer Centre (PMCC) Matrix as well as relative output measurements based on daily acquired portal images (unit G). Dashed lines indicate time points of known service events. Both portal imager data and PMCC Matrix data show sensitivity to output changes on the order of 2% associated with service events. The sinusoidal pattern evident in the PMCC Matrix data is not evident in the portal imager data suggesting the effect is specific to the instrument.

A comparison of output measurements using the portal imager with that of the standard method using the PMCC Matrix over a period of approximately a year is seen in Fig. 6. No processing was done to either dataset. Dashed lines correspond to dates where service events occurred. As can be seen, both sets of measurements are sensitive to changes in output on the order of 2% that occurred at the time of the service events. When comparing the datasets, the ion chamber measurements show a seasonal variation, whereas the portal imager measurements do not show an apparent seasonal trend. Discussion of the implications of this data and the relative limitations of the independent check using the MV portal imager follow in the Discussion section of this manuscript.

### 3.D Dosimeter response to humidity

**Figure 7:**
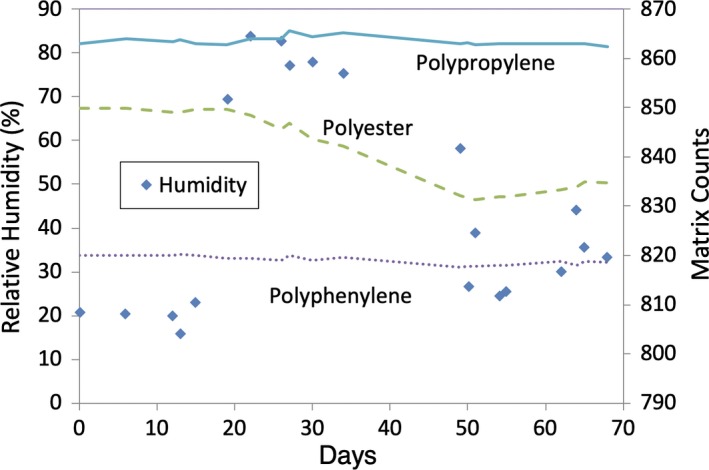
Matrix readout over time with a constant applied current source for three different channels utilizing a different capacitor type on each channel. The capacitor utilized in the original design is of the polyester type. A change in humidity was introduced at about day 20. While at high humidity conditions, a gradual decrease in Princess Margaret Cancer Centre Matrix readings is observed, to a maximum decrease on the order of 2%, on the channel utilizing the native polyester capacitor. Hysteresis in the change in readout is observed on the order of several weeks. The readings (counts) begin to increase gradually again when the humidity drops again near day 50.

In this controlled experiment, readout of the PMCC Matrix, using an input of a constant applied current source, was shown to markedly change under varied humidity conditions. Figure 7 shows this change to be on the order of 2% in readout occurring on the channel corresponding to the original polyester capacitor after exposure to several weeks of high relative humidity conditions. Readout was stable on the other two channels representing different capacitor types. Although the relative humidity increased from low to high conditions on the order of days, the readout change exhibited hysteresis on the order of weeks. Note that when humidity decreases again near day 50, the PMCC Matrix readings (counts) begin to observe a gradual increase.

## 4 DISCUSSION

As discussed in the methods, one of the potential causes for a variation in seasonal output is a measurement error in either temperature or pressure, where the error is both systematic and periodic over a long timescale. The datalogger recordings of temperature and pressure employed in this investigation showed excellent agreement with both the in room thermometer as well as the lab barometer utilized at our center for pressure–temperature corrections: only minimal discrepancies were observed, significantly less than what would be required to induce systematic variations on the order of 2% (peak to valley) in output measurements observed in Section 3.A. Thus, the above hypothesis was dismissed. Alternatively, it was suspected that the ambient conditions of the treatment room may be impacting either the dosimeter, the linear accelerator, or both in a cyclical way. Of the environmental conditions considered, humidity appeared to be the most likely factor related to the trends observed as humidity changes in the room were relatively large and seasonally variant in nature. In contrast, average temperature was controlled in the room to within approximately 2°C and pressure observed no significant systematic variation (mean) over time.

In the present work, it was also considered that daily open field EPID acquisitions utilized for daily QC might show detectable changes in signal value if the output of the accelerator were varying seasonally. The available EPID data were attractive because they are high frequency (daily), relatively independent of human setup errors and the device is assumed to be at equilibrium with the treatment unit. Analysis of open field images did indeed show a significant increase or decrease in signal corresponding to days where output was tweaked; yet, the signal variation did not show an identifiable periodic pattern on a long‐term scale, therefore suggesting that the periodic output changes observed were likely due to a change in sensitivity of readout of our daily QC dosimeter. The use of the MV portal imager data as a surrogate for output measurements in this paper may raise some concerns. At least one issue in utilizing the solid‐state detector (MV portal imager) data retrospectively is that no controls were in place to account for degradation of the panel, and that other factors that may impact the expected signal such as image lag or ghosting were not considered. It is important to note that the use of the MV portal imager data was utilized here explicitly to support or discredit whether the output changes observed were reflective of the changes occurring on the linear accelerator. To this extent (provided that the noise in the measurements is acceptably low), degradation of the panel, or any other factor that would vary the response of the panel, would only mask a seasonal trend from the accelerator if it carried its own canceling, anticorrelated humidity‐dependent variation. The absence of a seasonal trend in the MV portal imager data is therefore strong evidence that the periodic trends observed in historical output data are largely dosimeter dependent and not substantially due to a result of the output of the machines changing with ambient conditions.

It should be noted that though the impact of humidity changes on ion chamber measurements are expected to be of relatively minor consequence according to the literature,^7^ humidity can have adverse impacts on the other electrical components in the measurement device that may adversely impact the measurements. In particular, changes in leakage and capacitance may be related to changes in humidity and could potentially impact the readings to a higher order of magnitude than that deduced from changes in ion production alone.^8^ The addendum^6^ to task group 51 recommendations also notes that “The effect of extreme humidity values on equipment should also be considered (increased leakage, corrosion, etc.)”.

The sensitivity of the dosimeter to humidity and potential hysteresis effects were therefore investigated in the follow‐up controlled experimental investigation of the dosimeter (Sections 2.D and 3.D). In this controlled experiment, the readout of the dosimeter was found to be subject to humidity conditions as expected. When exposed to a change from low‐ to high‐humidity conditions, the signal changes on the PMCC Matrix were on the order of the observed changes in output of the linear accelerators (approximately 2%). Furthermore, significant hysteresis was noted where the response of signal change of the PMCC Matrix lagged behind more sudden humidity changes (on the order of weeks). This effect was consistent with the phase difference observed between humidity changes in the treatment unit and the measured output changes. This lag in response may be due to a long‐time necessary for the capacitor to approach equilibrium with the humidity of the surroundings. A slow response to changes would also explain why sudden spikes or drops in humidity are not met with changes in readings of the same order of the dosimeter.

The results of this study, therefore, provide very strong evidence that the seasonal changes observed do not reflect actual machine output changes to a substantial degree, but rather reflect the impact of humidity on the readout electronics of the measurement device for daily QC. Specifically, the native capacitor utilized in the onboard electronics was found to be particularly susceptible to humidity changes over time. We recall that the amplitude and phase of the output variations were consistent across linear accelerators despite presumably significant design differences, such as different monitor unit chamber designs (i.e., sealed vs unsealed). Thus, identification of the measurement device as the root cause of the seasonal output changes is logically coherent with the observations since the QC equipment is an underlying commonality between machines of different make and model.

It should be noted that the ion chamber‐based PMCC Matrix dosimeter is cross calibrated on an annual basis during the application of the TG 51 protocol. In addition, our QC program includes monthly farmer chamber measurements in solid water to monitor accelerator output. In our institution, the decision to adjust linac output was based on the review of the daily and monthly output measurements. Applications of TG 51 were also performed when disagreement between daily and monthly output was observed and sometimes resulted in recalibration of the PMCC Matrix dosimeter. Monthly ion chamber‐based output verification (not shown) did not demonstrate obvious seasonal variations. Due to the limited test frequency of these measurements and because they were subject to potential systematic uncertainty specific to this type of measurement (e.g., solid‐water temperature equilibrium and user‐dependent setup) without a method to reliably isolate such uncontrolled factors retrospectively, we did not have confidence that this data could adequately support or negate underlying trends in output, and therefore, it was not rigorously evaluated.

In light of the findings in this study, several courses of action could be implemented to manage or eliminate the impact of humidity on the measurement device output. As also suggested by Hossain,^12^ an immediate one could be to ensure that baseline measurements occur during time intervals corresponding to the mean of the output measurements in order to avoid false flags of machine tolerance being exceeded. Retrospective review suggests that, under that condition, approximately 27 service events, representing 63% of the events observed, may not have been triggered given that the tolerance would not have been approached during daily QC. However, this approach is not completely satisfactory in that it would still suggest accepting inaccuracies on the order of 1% in the measurement. Derivation of an empirically based correction factor for humidity may also be considered, though the hysteresis observed in readout changes may confound this approach. The identification of capacitors that are stable under marked humidity changes suggests that the best solution is modification of the relevant hardware. Alternative instruments for daily quality control could also be considered (as suggested by the use of the MV portal imager in this investigation).

As mentioned in the introduction, at least one other center has also reported seasonal variations in output; it is interesting to note that the standard quality control measurement tool used at that center for output constancy checks was similar in design to the system utilized in this study in the sense that all auxiliary electronics (e.g., electrometer) are integrated into a single unit. The present analysis also considered the same models and make of accelerator as utilized in that study, excluding differences in manufacturing as a likely cause. Although it was suggested by Hossain that the observed changes were likely a combination of effects impacting both the accelerators and the quality control dosimeters, this study supports the effect to be instrument specific and that the linear accelerators themselves are relatively stable to changes in relative humidity.

Finally, the trends observed in daily measurements also support the argument for a move from conventional quality assurance paradigms (e.g., action levels based on measurement outcomes over short timescales) towards more sophisticated approaches based on longer periods. Implementing a process control theory approach such as proposed by Sanghangthum et al. would, for example, be better suited for detecting gradual systematic variations such as those observed here.^13^

## 5 CONCLUSIONS

The results of this study indicate that the ion chamber‐based device used in this study for quality control of daily accelerator output exhibits seasonal variations that are humidity dependent. The data do not support the linear accelerators employed at our center having a significant response to humidity conditions or other environmental factors. Variations observed at this center are similar in nature to those reported elsewhere and suggest that there may be other commercially available devices that may be subject to similar changes as a response to environmental factors.

## CONFLICT OF INTEREST

No conflicts of interest.
